# Temperature management in acute type A aortic dissection treatment: deep vs. moderate hypothermic circulatory arrest. Is colder better?

**DOI:** 10.3389/fcvm.2024.1447007

**Published:** 2024-09-27

**Authors:** Hend Abdulwahab Muftah Abdulwahab, Alish Kolashov, Assad Haneya, Hannes Klump, Ajay Moza, Mohamad Fateh Arab, Mohammed Shoaib, Rashad Zayat, Mohammad Amen Khattab

**Affiliations:** ^1^ Department of Transfusion Medicine, Faculty of Medicine, RWTH University Hospital Aachen, RWTH Aachen University, Aachen, Germany; ^2^ Department of Cardiothoracic Surgery, Heart Centre Trier, Barmherzigen Brüder Hospital, Trier, Germany; ^3^ Department of Cardiac Surgery, Faculty of Medicine, RWTH University Hospital Aachen, RWTH Aachen University, Aachen, Germany; ^4^ Dr. Arab Cardiology Clinic, Jeddah, Saudi Arabia; ^5^ Department of Cardiac Surgery, Klinikum Links der Weser, Bremen, Germany; ^6^ Department of Thoracic Surgery, Faculty of Medicine, RWTH University Hospital Aachen, RWTH Aachen University, Aachen, Germany

**Keywords:** hypothermic circulatory arrest, acute type a aortic dissection, deep hypothermia, aortic surgery, moderate hypothermia

## Abstract

**Introduction:**

The impact of different degrees of hypothermia in patients undergoing type A aortic dissection (TAAD) repair remains controversial. The purpose of this study was to compare the clinical outcomes of patients who received deep hypothermic circulatory arrest (DHCA) (<20°C) and those of patients who received moderate hypothermic circulatory arrest (MHCA) (20–28°C).

**Methods:**

Between January 2011 and December 2020, 143 patients underwent surgical treatment for TAAD with CA and unilateral antegrade selective cerebral perfusion (uSCP). In this retrospective analysis, we evaluated the clinical outcomes of 143 individuals (103 who received DHCA vs. 40 who received MHCA). The primary outcome was the composite of major events (CMEs) including delirium, acute kidney injury (AKI), and in-hospital mortality. The secondary outcomes were overall mortality, bleeding, rethoracotomy, and length of intensive care unit (ICU) stay, among other things.

**Results:**

Compared with the MHCA group, the DHCA group presented a greater incidence of postoperative complications, as follows: AKI (26 (25.2%) vs. 3 (7.5%), *p *= 0.020), delirium (23 (22.3%) vs. 2 (5%), *p *= 0.014), re-exploration rate (21 (20.4%) vs. 2 (5.0%), *p *= 0.024), and prolonged intensive care unit (ICU) stay (7.8 (4.4, 14.1) vs. 5.7 (2.4, 10) days, *p *= 0.019). The median cardiopulmonary bypass time (255 (210, 280) vs. 210 (190, 251) min, *p *= 0.010) and median cross-clamp time (140 (110, 180) vs. 125 (100, 160) min, *p *= 0.023) were significantly longer in the DHCA group. The German Registry for Acute Aortic Dissection Type A (GERAADA) score was significantly higher in the MHCA group (22.7 ± 9.1 vs. 19 ± 7.2, *p *= 0.012). The adjusted odds ratio for CME in the MHCA group was 0.78 (95% CI: 0.52–1.17, *p *= 0.001). The use of MHCA demonstrated a protective effect on reducing postoperative delirium (OR: 0.28, 95% CI: 0.14–0.46, *p *< 0.01) and postoperative AKI (OR: 0.29, 95% CI: 0.14–0.49, *p *< 0.01). Overall survival after two years did not differ between the two groups (log-rank, *p *= 0.16).

**Conclusion:**

The principal findings of our study indicate that DHCA elevates the risk of postoperative AKI and delirium. As a result, the duration of hospitalization and intensive care unit stay was markedly extended. Consequently, MHCA should be favored over DHCA when the clinical circumstances permit, since DHCA remains a secure alternative in intricate dissection instances.

## Introduction

Aortic dissection is a rare but life-threatening medical condition with a significant mortality rate. Despite early detection, sophisticated surgery, and improved perioperative treatment, the in-hospital death rate remains high among patients suffering from acute type A aortic dissection (ATAAD) (1, 2). Nevertheless, compared with other therapeutic options, surgery results in the highest survival rate after ATAAD. However, compared with other cardiovascular procedures, treatment of ATAAD with surgery has the highest mortality rate. The primary causes of mortality are predominantly neurological, cardiac, ischemic, and hemorrhagic (3–5). The choice of appropriate surgical strategy has remained challenging in recent decades because of the substantial susceptibility of brain tissue to ischemia and the high risk of embolization during aortic repair. Hypothermia can sufficiently reduce cerebral metabolic demand to allow reasonable periods of circulatory arrest (5, 6). A lower internal temperature ensures reduced metabolic activity (7, 8). However, lower temperatures during hypothermic circulatory arrest (HCA) require longer rewarming times, require longer cardiopulmonary bypass (CPB) times, and are associated with increased risks of CPB-related complications (9–11). Our study aimed to investigate the perioperative morbidity and mortality rates associated with implementing deep hypothermic circulatory arrest (DHCA) vs. moderate hypothermic circulatory arrest (MHCA).

## Materials and methods

### Patient collection and study protocol

The study was conducted at the cardiothoracic surgery department of the University Hospital of RWTH Aachen in Germany. A review of our clinical electronic database from January 2011 to January 2020 revealed that 166 consecutive patients underwent surgical treatment for ATAAD. Twenty-three patients were excluded according to the following exclusion criteria: mild hypothermic circulatory arrest (*n* = 7), profound hypothermic circulatory arrest (*n* = 5), and death before establishing CPB due to major bleeding (*n* = 11). The patients were divided into two groups and received DHCA (*n* = 103) between 14.1–20°C or MHCA (*n* = 40) between 20.1–28°C (6). We conducted our study with retrospective patient data collected from our institutional database. In accordance with the STROBE criteria and the Declaration of Helsinki, approval was authorized by the Research Ethics Committee at RWTH University Aachen, Germany (EK 20-003). The ethics board waived the requirement for informed consent, considering the retrospective nature of the study.

### Definitions of outcomes

The primary endpoint of our study was a composite of major events (CME), which included in-hospital mortality, postoperative delirium, and acute kidney injury. In-hospital mortality was defined as death that occurred during the same hospital stay when the operation was performed. Acute kidney injury (AKI) was defined by the Kidney Disease Improving Global Outcomes criteria (9). the secondary endpoints included the following: the incidence of newly developed permanent neurological deficit (PND), which was confirmed by a cranial CT scan; temporary neurological deficit (TND), which was defined as the occurrence of reversible delirium, agitation, disorientation, or motoric deficit that was resolved prior to discharge (as evidenced by a normal CT scan); postoperative sepsis, pneumonia, bleeding, rethoracotomy, length of ICU-stay and total length of hospital stay.

### Surgical technique and perioperative standard

The ATAAD diagnosis was confirmed by contrast-enhanced CT. In accordance with our hospital standards, all procedures were performed emergently by an experienced surgical team. Our monitoring included a five-lead ECG, pulse oximetry, near-infrared spectroscopy (NIRS), transesophageal echocardiography (TEE), and urinary bladder and nasopharyngeal temperature monitoring. The patients were divided into the DHCA and MHCA groups according to nasopharyngeal temperature. Arterial lines were placed unilaterally in the femoral artery and in both radial arteries. General anesthesia was induced with a combination of propofol, sufentanil, and rocuronium. A midline sternotomy was performed immediately. Prior to the initiation of CPB, intravenous (i.v.) heparin was administered in a weight-adjusted dose until the activated coagulation time (ACT) reached at least 450 s Subsequently, cannulation was performed. The variations in arterial cannulation sites were arranged as follows: the right subclavian artery with an artificial vessel graft via an end-to-side anastomosis, the femoral artery, the left ventricle (LV), or the ascending aorta. Venous cannulation was performed in the femoral vein or the right atrium. After the initiation of CPB, the patients were cooled to establish targeted hypothermic circulatory arrest (HCA). After cross-clamping of the aorta, the heart was arrested with 4–6°C Bretschneider solution (CUSTODIOL®) following our clinical protocol. Moreover, 1,000 mg of methylprednisolone was intravenously administered during the anesthesia induction process, followed by 500 mg of sodium thiopental after the initiation of CPB for neuroprotection. Furthermore, ice packs were used to cool the head. Unilateral antegrade selective cerebral perfusion (uSCP) was established by directly cannulating the innominate artery with a 9F catheter. The cannula was used to initiate uSCP at a rate of 10–12 ml/kg/min. The target pressure ranged from 50 to 60 mmHg, and the perfusate temperature ranged from 20 to 28°C.

In the past ten years, there has been a shift from using DHCA to using MHCA during ATAAD repair at our institution. The choice between DHCA and MHCA, and the use of uSCP, was based on the surgeon's best performance and expertise and was influenced by various criteria such as age, preoperative renal function, aortic disease, and the level of complexity involved in the intended arch reconstruction. This is in accordance with the EACTS/STS guidelines (12), as there is a class IIa recommendation with the following statement: A target hypothermic circulatory arrest temperature should be determined based on the anticipated extent of repair, expected duration of lower body HCA and presence of preoperative malperfusion.

### Statistical analysis

Means and standard deviations are used to present continuous variables, while percentages and absolute numbers are used to present categorical variables. The R Studio interface version 4.0.3 (RStudio Team, Boston, MA) was used for all analyses, alongside with the jamovi project (2020) (jamovi computer software, version 2.3.8; JAMovi.org), which is available at https://www.jamovi.org/. The Kolmogorov‒Smirnov test was used to assess the distribution of continuous data. The data that followed a normal distribution are presented as mean ± standard deviation (SD) and were analyzed using the Student's *t*-test. The median and interquartile range were utilized to characterize data that did not follow a normal distribution, while the Mann‒Whitney *U*-test was used for comparison. The categorical variables were analyzed using either the chi-square test or Fisher's exact test. Additionally, to calculate the adjusted odds ratio (OR) of postoperative complications after MHCA compared to those after DHCA, variables with a *p *< 0.05 in the univariable analysis were entered into a multivariable logistic regression model (backward stepwise logistic regression) with adjustments for confounders, including preoperative malpersion and the German Registry for Acute Aortic Dissection Type A (GERAADA) score (13). Given the limited sample size of the MHCA group and to mitigate potential confounding variables, we employed the propensity scores (PS) methodology. Logistic regression analysis was employed to determine the propensity score, and a closest neighbor matching approach was utilized at a 1:1 ratio. The matching caliber was established at 0.2 times the standard deviation of the logit score. Covariates comprised age, gender, GERAADA score, and malperfusion status. Given the limited sample size of the MHCA group and to mitigate potential confounding variables, we employed the propensity scores (PS) methodology. Logistic regression analysis was employed to determine the propensity score, and a closest neighbor matching approach was utilized at a 1:1 ratio. The matching caliber was established at 0.2 times the standard deviation of the logit score. Covariates comprised age, gender, GERAADA score, and malperfusion status. After matching we used the treatment effect analysis from STATA. eteffects estimates the average treatment effect (ATE), the average treatment effect on the treated (ATET), and the potential-outcome means (POMs) from observational data when treatment assignment is correlated with the potential outcomes. It allows for continuous, binary, count, fractional, and nonnegative outcomes and requires a binary treatment (14). Kaplan–Meier survival curves were generated, and the log-rank test was used to assess linear trends. All *p* values were rounded to three decimal places or are presented as a number including at least one nonzero digit. A *p*-value of 0.05 or lower indicates statistical significance.

## Results

### Pre- and perioperative comparisons

After screening a total of 166 patients, 23 were excluded. The final group consisted of 143 patients, including 103 (72%) who underwent aortic surgery with DHCA and 40 who underwent aortic surgery with MHCA (28%) (Table 1). The DHCA and MHCA groups had mean ages of 61.1 ± 12.2 and 60.9 ± 14.5 years, respectively, and included 36 (35%) and 11 (27%) females, respectively. The GERAADA score was significantly higher in the MHCA group than in the DHCA group (22.7 ± 9.1% vs. 19 ± 7.2%, *p *= 0.012). The preoperative occurrence of peripheral and coronary malperfusion did not differ between the groups, whereas the incidence of preoperative visceral malperfusion was significantly higher in the MHCA group than in the DHCA group (14 (35%) vs. 16 (15.7%), *p *= 0.021, respectively) (Table 1). The distribution of cannulation sites did not differ between the two groups (Table 1). The median body temperature was 18°C (18, 19) in the DHCA group and 24°C (21.5, 25) in the MHCA group (*p *< 0.001).

**Table 1:** Patient's demographics and intraoperative data.

	DHCA (*n* = 103)	MHCA (*n* = 40)	*p* values
Patient demographics			
Female, *n* (%)	36 (35)	11 (27)	0.434
Mean age, years	61.1 ± 12.2	60.9 ± 14.5	0.905
Hypertension, *n* (%)	54 (52.4)	19 (47.5)	0.710
COPD, *n* (%)	7 (6.8)	3 (7.5)	1.000
Prior cardiac disease, *n* (%)	11 (10.5)	9 (22.5)	0.104
Coronary artery disease, *n* (%)	19 (18.4)	8 (20.0)	0.816
Acute coronary syndrome, *n* (%)	6 (5.8)	2 (5.0)	1.000
Marfan syndrome, *n* (%)	1 (1.0)	1 (2.5)	0.483
GERAADA score%	19 ± 7.2	22.7 ± 9.1	**0**.**012**
Peripheral malperfusion *n* (%)	17 (16.5)	11 (27.5)	0.210
Coronary malperfusion *n* (%)	17 (16.5)	7 (17.5)	1.000
Visceral malperfusion *n* (%)	16 (15.7)	14 (35)	**0**.**021**
Preoperative CPR *n* (%)	3 (2.91)	2 (5)	0.651
Preoperative creatinine mg/dl	1.6 ± 0.8	1.5 ± 0.6	0.106
Intraoperative variations			
Dissection of the ascending aorta alone, *n* (%)	8 (7.8)	1 (2.6)	0.444
Dissection including aortic arch, *n* (%)	4 (3.9)	1 (2.5)	1.000
Dissection including brachiocephalic trunk, *n* (%)	61 (59.2)	20 (50.0)	0.351
Dissection reaching distal iliac vessels, *n* (%)	28 (27.2)	18 (48.6)	**0**.**024**
Preoperative creatinine mg/dl	1.4 ± 0.8		
Ascending aorta cannulation, *n* (%)	22 (21.6)	15 (37.5)	0.320
Subclavian artery cannulation, *n* (%)	40 (45.6)	13 (32.5)	0.564
LV apex cannulation, *n* (%)	4 (3.9)	0 (0.0)	0.576
Femoral artery cannulation, *n* (%)	37 (35.9)	12 (30)	0.564
uSCP, *n* (%)	90 (87.3)	32 (80)	0.296
Femoral vein cannulation, *n* (%)	33 (32.1)	20 (50)	0.054
Right atrium cannulation, *n* (%)	70 (67.9)	20 (50)	0.054
Supra-coronary ascending aorta replacement, *n* (%)	28 (27.2)	20 (50)	**0**.**017**
Bentall procedure, *n* (%)	19 (18.4)	9 (22.5)	0.318
ascending aorta replacement + partial arch replacement, *n* (%)	10 (9.7)	7 (17.5)	0.246
Total aortic arch replacement: Island reinsertion technique, *n* (%)	28 (27.2)	3 (7.5)	**0**.**012**
Total aortic arch replacement: Debranching, *n* (%)	18 (17.4)	1 (2.5)	**0**.**024**
CPB time, min	255 (210, 280)	210 (19, 251)	**0**.**010**
Cross-clamp time, min	140 (110, 180)	125 (100, 160)	**0**.**023**
Cardiac arrest time, min	47.6 ± 17.6	41.3 ± 16.0	0.336
Median body temperature °C	18 (18, 19)	24 (21.5, 25)	**<0**.**001**

Normal distributed data are presented as means ± SD; non-normal distributed data are presented as median (25th, 75th percentiles). °C, grad Celsius; COPD, chronic obstructive pulmonary disease; CPB time, Cardiopulmonary bypass time; DHCA, deep hypothermic circualtoryarrest; GERAADA, German Registry for acute aortic dissection type A; LV, left ventricle; MHCA, moderate hypothermic circualtoryarrest; uSCP, Unilateral selective antegrade cerebral perfusion; **Bold** entries indicate significance.

Patients in the MHCA group had significantly more isolated supracoronary ascending aorta replacements (20 (50%) vs. 28 (27.9%); *p *= 0.024). The number of patients who underwent Bentall procedures and ascending aorta replacement + partial aortic arch replacement was similar between the two groups (Table 1). Total arch replacement, either via reimplantation of an island of the aortic wall containing the ostia of the innominate artery (IA), left carotid artery (LCA), or left subclavian artery (LSA), was performed significantly more often under DHCA conditions (28 (27.2%) vs. 3 (7.5%); *p *= 0.012) or using the anatomic branch technique (18 (17.4%) vs. 1 (2.5%); *p *= 0.024) (Table 1).

### Postoperative complications

**Figure 1:**
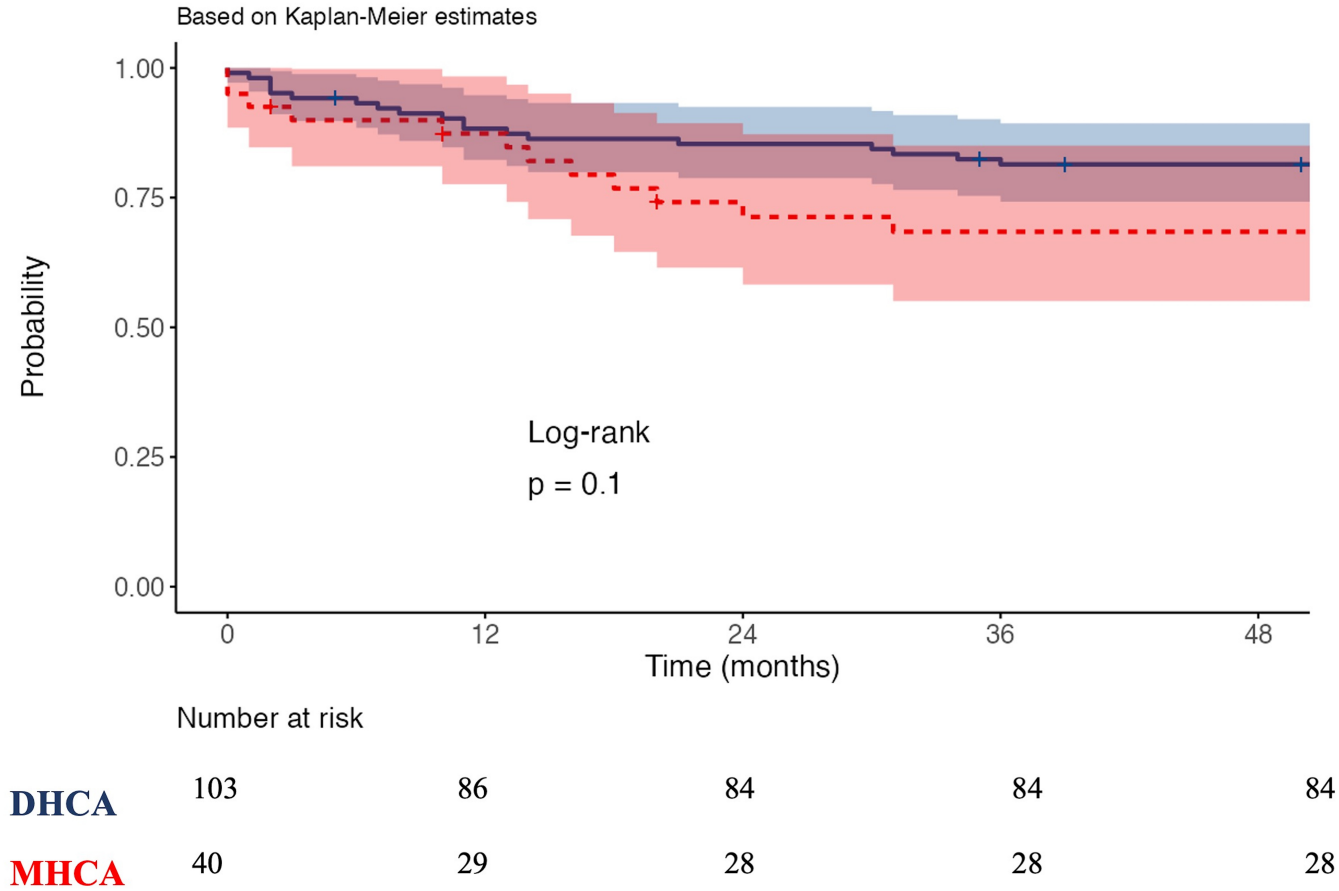
Kaplan-Meier survival curves. DHCA: Deep hypothermia circulatory arrest, MHCA: moderate hypothermia circulatory arrest.

The CPB duration and cross-clamp time were significantly prolonged in the DHCA group compared to the MHCA group: CPB time (255 (210, 280) vs. 210 (190, 251) min, *p = *0.010) and cross-clamp time (140 (110, 180) vs. 125 (100, 160) min, *p = *0.023) (Table 2). The incidence of bleeding requiring rethoracotomy was significantly greater in the DHCA group than in the MHCA group (21 (20.4%) vs. 2 (5.0%); *p *= 0.024). The frequency of acute kidney injury (AKI) with the need for dialysis was also significantly higher in the DHCA group than in the MHCA group (26 (25.2%) vs. 3 (7.5%), *p *= 0.020), as well as the incidence of postoperative delirium (23 (22.3%) vs. 2 (5%), *p *= 0.014) (Table 2). Patients in the DHCA group had substantially longer mechanical ventilation times than patients in the MHCA group (44.0 (7, 246) vs. 8.5 (0.8, 91.8) hr., *p *= 0.012). Consequently, the DHCA group exhibited prolonged intensive care unit (ICU) stays (7.8 (4.4, 14.1) vs. 5.7 (2.4, 10) days, *p *= 0.019) as well as longer entire hospital lengths of stay compared with the MHCA group (16.2 (10.1, 29.9) vs. 13.2 (8, 18.6) days, *p *= 0.012). In terms of postoperative ischemic or hemorrhagic stroke, no significant difference was noted between the two groups, and in-hospital mortality did not differ between the two groups (17 (16.5%) vs. 11 (27.5%); *p *= 0.212). The Kaplan‒Meier curve after the 2-year follow-up revealed no significant difference in survival between patients who received DHCA and those who received MHCA (Figure 1). We did not find any correlation between the HCA time and postoperative outcomes.

**Table 2:** Postoperative outcomes.

	DHCA (*n* = 103)	MHCA (*n* = 40)	*p* values
Re-exploration due to bleeding, *n* (%)	21 (20.4)	2 (5.0)	**0**.**024**
Pericardial effusion, *n* (%)	11 (10.7)	1 (2.5)	0.179
Cardiac tamponade, *n* (%)	3 (2.9)	0 (0.0)	0.560
Acute kidney injury with dialysis, *n* (%)	26 (25.2)	3 (7.5)	**0**.**020**
Acute liver failure, *n* (%)	15 (14.6)	4 (10.0)	0.589
Delirium, *n* (%)	23 (22.3)	2 (5.0)	**0**.**014**
Deep sternal wound infection, *n* (%)	5 (4.9)	0 (0.0)	0.322
Pneumonia, *n* (%)	38 (38.0)	9 (23.7)	0.159
Atrial fibrillation, *n* (%)	38 (36.9)	15 (37.5)	1.000
Ischemic stroke, *n* (%)	19 (18.4)	7 (17.5)	1.000
Hemorrhagic stroke, *n* (%)	3 (2.9)	3 (7.5)	0.349
Mechanical ventilation time, hr.	44.0 (7, 246)	8.5 (0.8, 91.8)	**0**.**012**
ICU stay, days	7.8 (4.4, 14.1)	5.7 (2.4, 10)	**0**.**019**
Hospital LOS, days	16.2 (10.1, 29.9)	13.2 (8, 18.6)	**0**.**012**
In-hospital mortality	17 (16.5)	11 (27.5)	0.212
2-years mortality	19 (18.4)	12 (30.0)	0.201

Normal distributed data are presented as means ± SD; non-normal distributed data are presented as median (25th, 75th percentiles). ICU, intensive care unit; LOS, length of stay.

Bold indicate significance difference.

### Regression analysis

To identify independent predictors of CME, we conducted univariable logistic analyses, which were subsequently followed by stepwise backward multivariable logistic analyses using the GERAADA score and malperfusion status as covariates to calculate the adjusted odds ratio. Table 3 presents the adjusted multivariable logistic analyses. The adjusted odds ratio for CME in the MHCA group was 0.78 (95% CI: 0.52–1.17, *p *= 0.001). The use of MHCA demonstrated a protective effect on reducing postoperative delirium (OR: 0.28, 95% CI: 0.14–0.46, *p *< 0.01) and postoperative AKI (OR: 0.29, 95% CI: 0.14–0.49; *p *< 0.01).

**Table 3:** Multivariable logistic regression adjusted to GERAADA score and malperfusion.

CMO as dependent variable				95% Confidence interval		Z	*P*
Predictors	Estimate	SE	Odds ratio	Lower	Upper		
GERAADA	0.05	0.03	1.05	0.99	1.12	1.66	0.098
Peripheral malperfusion	−0.35	0.49	0.71	0.27	1.84	−0.71	0.477
Coronary malperfusion	0.00	0.55	1.00	0.34	2.92	0.01	0.995
Visceral malperfusion	−0.82	0.57	0.44	0.14	1.35	−1.44	0.150
DHCA (MHCA-DHCA	−0.25	0.21	0.78	0.52	1.17	−1.20	0.001
Delirium as dependent variable				95% Confidence interval		Z	*P*
Predictors	Estimate	SE	Odds ratio	Lower	Upper		
GERAADA	−0.04	0.04	0.96	0.89	1.04	1.04	0.285
Peripheral malperfusion	0.23	0.59	1.26	0.39	4.04	4.04	0.700
Coronary malperfusion	1.04	0.65	2.84	0.80	10.05	10.05	0.106
Visceral malperfusion	1.11	0.68	3.02	0.79	11.55	11.55	0.106
DHCA (MHCA-DHCA)	−1.26	0.25	0.28	0.17	0.46	0.46	<.001
AKI as dependent variable				95% Confidence interval		Z	*P*
Predictors	Estimate	SE	Odds ratio	Lower	Upper		
GERAADA	−0.09	0.04	0.91	0.84	0.99	−2.23	0.026
Peripheral malperfusion	0.93	0.57	2.53	0.83	7.70	1.63	0.103
Coronary malperfusion	−0.72	0.85	0.49	0.09	2.55	−0.85	0.395
Visceral malperfusion	0.86	0.68	2.37	0.62	9.00	1.27	0.205
DHCA (MHCA-DHCA)	−1.23	0.26	0.29	0.18	0.49	−4.72	<.001

AKI, acute kidney injury; GERAADA, German Registry for acute aortic dissection type A; SE, standard error; Z, linear combination of predictor variables and their corresponding coefficients.

### Subanalysis comparing patients with uSCP and those without uSCP

Supplementary Table S1 summarizes the comparison of preoperative characteristics and postoperative outcomes between patients who underwent ATAAD repair with or without uSCP. The incidence of ATAAD only in the ascending aorta was significantly greater in patients who underwent surgery without uSCP (8 (57.1%) vs. 1 (0.8), *p *< 0.001). The most common operation performed without uSCP was supracoronary replacement of the ascending aorta (13 (92.8%) vs. 35 (27.1), *p *= 0.001). The CPB time and cross-clamp time were significantly shorter in the group without uSCP than in the uSCP group (CPB: 208.8 ± 29.3 min. vs. 245.8 ± 53.5 min, *p *= 0.012; cross-clamp time: 117.9 ± 36.2 min vs. 145.2 ± 38.2 min, *p *= 0.012, respectively). There was no significant difference in postoperative complications between the two groups (Supplementary Table S1). In-hospital mortality did not differ between the two groups, but two-year survival was better in the uSCP group (log-rank, *p *= 0.029) (Supplementary Figure S1).

### Propensity matching and the treatment effect analysis

**Figure 2:**
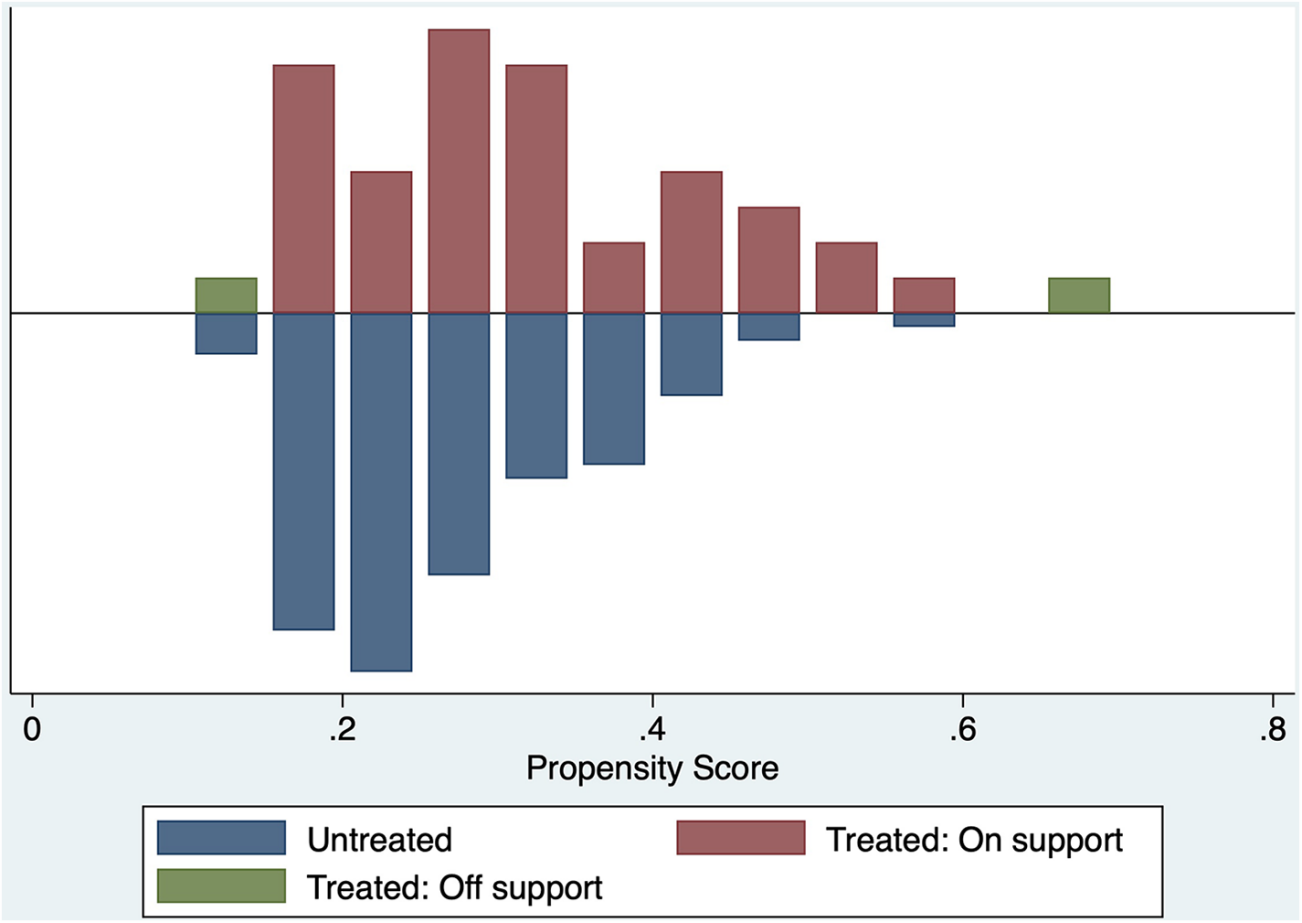
Propensity matching balance.

After we performed the propensity matching and matched the 40 patients from the MHCA group to 40 patients from the DHCA group. Figure 2 demonstrate the balance in the matching. After matching we compared both groups in terms of CME using the treatment effect analysis (Table 4). The treatment effect estimation showed a significant difference between DHCA vs. MHCA in the incidence of CME (coefficient:−.493, 95%-CI: −.633 to −.353, *p *< 0.01).

**Table 4:** Treatment effect estimation.

CME	Coef.	St.Err.	t-value	*p*-value	[95% Conf	Interval]	Sig
DHCA vs. MHCA	−.493	.071	−6.92	<0.01	−.633	−.353	***
Mean dependent var		0.441	SD dependent var			0.498	

DHCA, deep hypothermic cardiac arrest; MHCA, moderate hypothermic cardiac arrest; St. Err, standard error.

*** *p < .01*.

## Discussion

Considering the brain's susceptibility to hypoxemia and the long-standing surgical experience in neuroprotection after aortic surgical treatment based on HCA, numerous surgeons worldwide aim to minimize the oxygen demand with more extensive hypothermia (7).

Compared with the DHCA group, the MHCA group exhibited better preservation of renal function and fewer cases of AKI. DHCA patients not only had worse kidney function parameters but also required external renal replacement therapy substantially more often (15). In contrast to our findings regarding the higher incidence of AKI in the DHCA group, Tsai JY et al. (16) analyzed data from 221 consecutive patients who underwent aortic arch replacement and found no difference in the incidence of AKI between patients who received DHCA and those who received MHCA. Tsai JY et al. (16) also reported that temperature did not notably affect the need for blood transfusions or rethoracotomy, which contrasts with our findings. In accordance with our results, Zhou et al. (17) investigated the outcomes of 533 patients who underwent total arch replacement combined with a frozen Elefant trunk, either with MHCA or DHCA. Zhou and colleagues reported that the incidence of AKI was higher in the DHCA group and was associated with higher mortality (17).

On the other hand, Wu et al. (18) analyzed data from 438 patients with ATAAD who were treated either with DHCA or MHCA. Similar to our findings, they reported that MHCA has a protective effect on reducing postoperative AKI and in-hospital mortality. In accordance with our findings, they also reported that patients treated with DHCA had longer ICU stays and hospital stays (18).

Cao et al. (19) performed a meta-analysis to analyze the effect of DHCA vs. MHCA in aortic arch surgery on postoperative renal function. They (19) included a total of 14 observational studies with 4,142 patients, and similar to our findings, they reported that compared with DHCA, MHCA reduces the incidence of renal failure and the need for renal replacement.

Li et al. (20) proposed that hypothermia during circulatory arrest techniques may serve as an independent risk factor for acute kidney injury (AKI). In accordance with our findings, independent investigations by other research groups have also shown a correlation between AKI and DHCA (21, 22). Li et al. (20) found that in the event of post-surgical AKI. Mechanistically, increased bradykinin, resulting from a temperature decrease during HCA, inhibited the Nrf2-xCT pathway and heightened oxidative stress, culminating in postoperative AKI.

The larger temperature span allows more re-warming, consequently increasing the total CPB time, leading to detrimental multifactorial coagulopathy (23, 24). There was no discernible decrease in the incidence of cardiac tamponade or pericardial effusion in the MHCA group, whereas the causes of surgical bleeding were evenly prevalent between the two groups. However, the DHCA group had a greater re-exploration rate due to major bleeding during the ICU stay. Re-exploration for excessive bleeding after cardiothoracic surgery is associated with worse outcomes, including significantly higher postoperative mortality and morbidity, and should be avoided (25).

Additionally, the study revealed that individuals in the DHCA group required prolonged mechanical ventilation. One significant factor may be the superior vascular constriction in the peripheral body areas at lower temperatures, leading to continuous cold blood return, resulting in prolonged hypothermia during the initial hours of the patient's stay in the ICU. In accordance with our findings, Leshnower et al. (26) analyzed 288 patients who underwent ATAAD and compared outcomes between patients who received DHCA and those who received MHCA. They reported a reduction in postoperative mechanical ventilation time and ICU stay in the MHCA group. Leschnower et al. also reported an association between prolonged CPB time in the DHCA group and postoperative pulmonary dysfunction (26).

A recent study by Liu et al. (27) suggested that a prolonged postoperative ICU stay was associated with emergency surgery, preoperative urea nitrogen levels, and CPB time. Compared with the regular ICU stay group, the prolonged ICU stay group experienced a significantly higher incidence of adverse events, including tracheotomy, reintubation, 72 h of tracheal extubation after surgery, 12 h of consciousness recovery after surgery, ICU re-entry, and irregular discharge (27).

In contrast to our results, Hameed et al. (28) conducted a large meta-analysis that included 26,968 patients from 68 studies. They found that antegrade cerebral perfusion and retrograde cerebral perfusion were associated with substantially lower postoperative stroke and operative mortality rates compared with DHCA. They also reported that the duration of circulatory arrest was linked to the neuroprotective benefits of antegrade and retrograde cerebral perfusion when compared with deep hypothermic circulatory arrest. In our study, we did not identify any correlation between the HCA time and postoperative outcomes.

Another crucial aspect is the higher occurrence of delirium among the DHCA group. Postoperative delirium is a common complication that may occur after cardiac surgery. It extends the duration of the patient's ICU stay, poses a risk to the safety of both the patient and medical staff, and causes considerable emotional distress to patients and their relatives. While profound hypothermia might reduce metabolic demand *in situ*ations where energy substrates are scarce, it can also have several negative repercussions. Both human and animal investigations have provided evidence indicating that neuronal damage is associated with profound hypothermia followed by rewarming alone (29). Our findings regarding the lower incidence of postoperative delirium and ICU stay in the MHCA group are similar to the findings of Khaladj et al. and Tsai et al. (16, 30).

Prolonged CPB duration is a consistent independent factor associated with a higher occurrence of delirium (31–33). Additionally, the prolonged mechanical ventilation observed in the DHCA group is another factor that contributes to delirium (34).

Hughes et al. (35) studied 309 patients in a randomized single-blind trial [GOT ICE (Cognitive Effects of Body Temperature During Hypothermic Circulatory Arrest)] of patients undergoing arch surgery with HCA plus antegrade cerebral perfusion at 4 US referral aortic centers. Hughes et al. (35) found a notable regional postoperative alteration in cortical thickness, which wasdetected in 18 locations of focus, often within the 0.05–0.10 mm range. Most of these variations were bilaterally manifested in the inferior frontal and dorsolateral prefrontal cortices (35). These findings may give more insights information about the possible postoperative cognitive and neurological function, which can occur after DHCA or MHCA.

On the other hand, the lower temperature used in HCA did not provide any additional advantages in terms of neuroprotection. There was no increase in the occurrence of either ischemic or hemorrhagic stroke among individuals with less hypothermia. The overall incidence of other adverse effects was similar in both groups. These findings are consistent with those of Leshnower et al. and Tsai et al. (16, 26), who reported no significant differences between DHCA and MHCA with respect to adverse permanent neurological outcomes.

The findings of the present study strongly support the idea that the combination of SCP and MHCA provides adequate protection to the brain and internal organs of patients undergoing ATAAD repair, eliminating the need for severe hypothermia. A prospective, randomized study comparing DHCA and MHCA with uSCP could help determine if DHCA offers any benefits over MHCA in terms of patient outcomes. While this study and others have reported favorable results with MHCA for treating ATAAD, it is challenging to persuade surgeons to subject patients to the additional CPB times and potential risks of DHCA, including multiorgan endothelial dysfunction (36). Thus, comprehensive retrospective assessments of alternative approaches will likely continue to shape circulatory therapy in acute type A aortic dissection surgery.

## Study limitations

This study was limited as it was a retrospective cohort study conducted at a single institution. Most patients who received surgery earlier in the research period were subjected to deep hypothermia, whereas those who underwent surgery more recently experienced moderate hypothermia. Therefore, it is plausible that perioperative care approaches have changed slightly over time, thereby biasing our results. However, the degree of uncertainty regarding the extent of aortic replacement and other concomitant procedures necessary to ensure patient survival is one of the most difficult aspects of repairing ATAAD. These decisions are frequently made intraoperatively after evaluating the aortic intima from the valve level to the left subclavian artery. Consequently, the creation of two homogeneous groups for comparing perfusion strategies is challenging due to the high degree of variability in the operations performed to repair ATAAD. Finally, we did not find any difference in the two years survival but due to the lack of follow-up information such as quality of life, dialysis and neurological findings, necessitates additional research to assess the advantages of moderate hypothermia during follow-up.

## Conclusion

The principal findings of our study indicate that DHCA elevates the risk of postoperative AKI and delirium. As a result, the duration of hospitalization and intensive care unit stay was markedly extended. Consequently, MHCA should be favored over DHCA when the clinical circumstances permit, since DHCA remains a secure alternative in intricate dissection instances.

## Data Availability

The original contributions presented in the study are included in the article/Supplementary Material, further inquiries can be directed to the corresponding authors.
