# Supplementary material for: Temperature management in acute type A aortic dissection treatment: deep vs. moderate hypothermic circulatory arrest. Is colder better?
Source: Front Cardiovasc Med. 2024 Sep 27;11:1447007. doi: 10.3389/fcvm.2024.1447007 (PMC11548057; doi:10.3389/fcvm.2024.1447007)
Supplement: Supplementary file 1 [file Datasheet1.docx]

**Supplementary materials:**

**Supplementary Table S1.**

|  | without uSCP | with uSCP | p |
| --- | --- | --- | --- |
| Total n (%) | 14 (9.8) | 129 (90.2) |  |
| Dissection including supra-aortal vessels n (%) | 2 (14.3) | 79 (61.2) | **0.001** |
| Dissection reaching the aortic arch n (%) | 4 (28.6) | 1 (0.77) | **0.003** |
| Dissection reaching distal iliac vessels n (%) | 0 (0.0) | 46 (35.6) | **0.005** |
| Dissection including brachiocephalic trunk, n (%) | 0 (0) | 81 (62.8) | **<0.001** |
| Dissection of the ascending aorta alone, n (%) | 8 (57.1) | 1 (0.8) | **<0.001** |
| Total aortic arch replacement: Island  re-insertion technique, n (%) | 0 (0.0) | 31 (24) | **0.040** |
| Bentall-Operation n (%) | 0 (0.0) | 28 (21.7) | 0.051 |
| ascending aorta replacement +  partial arch replacement, n (%) | 2 (14.3) | 15 (11.6) | 0.671 |
| Supra-coronary ascending aorta replacement, n (%) | 13 (92.8) | 35 (27.1) | **0.001** |
| DHCA n (%) | 1 (7.1) | 102 (79.1) | <0.001 |
| CPB time minutes | 208.8±29.3 | 245.8±53.5 | **0.012** |
| Cross clamp time minutes | 117.9±36.2 | 145.2±38.2 | **0.012** |
| Hypothermic circulatory arrest time minutes | 47.7±14.0 | 47.9±13.9 | 0.598 |
| AKI n (%) | 0 (0.0) | 29 (22.5) | 0.102 |
| Pneumonia n (%) | 3 (23.1) | 44 (35.2) | 0.568 |
| Postop ischemic stroke n (%) | 3 (21.4) | 23 (17.8) | 1.000 |
| Haemorrhagic stroke n (%) | 2 (14.3) | 4 (3.1) | 0.200 |
| Delirium n (%) | 0 (0.0) | 25 (19.4) | 0.149 |
| ICU stay days | 4.2±3.0 | 11.6±11.0 | **0.019** |
| Ventilation time min. | 31.5 (44.9) | 155.5 (241.4) | 0.058 |
| LOS days | 10.9±8.3 | 18.9±12.7 | **0.024** |
| Mortality n (%) | 6 (42.9) | 25 (19.4) | 0.092 |
| CME n (%) | 10 (71.4) | 80 (62.0) | 0.688 |
| In-hospital Mortality n (%) | 6 (42.9) | 22 (17.1) | 0.050 |

 AKI: Acute kidney injury, CME: composite majore adverse events, CPB: Cardiopulmonary bypass, DHCA: deep hypothermic circulatory arrest ICU: intensive care unit, LOS: length of stay

**Figure S1. Kaplan-Meier-Survival curve comparing patients who had acute aortic type A dissection with uSCP vs. without uSCP**

**
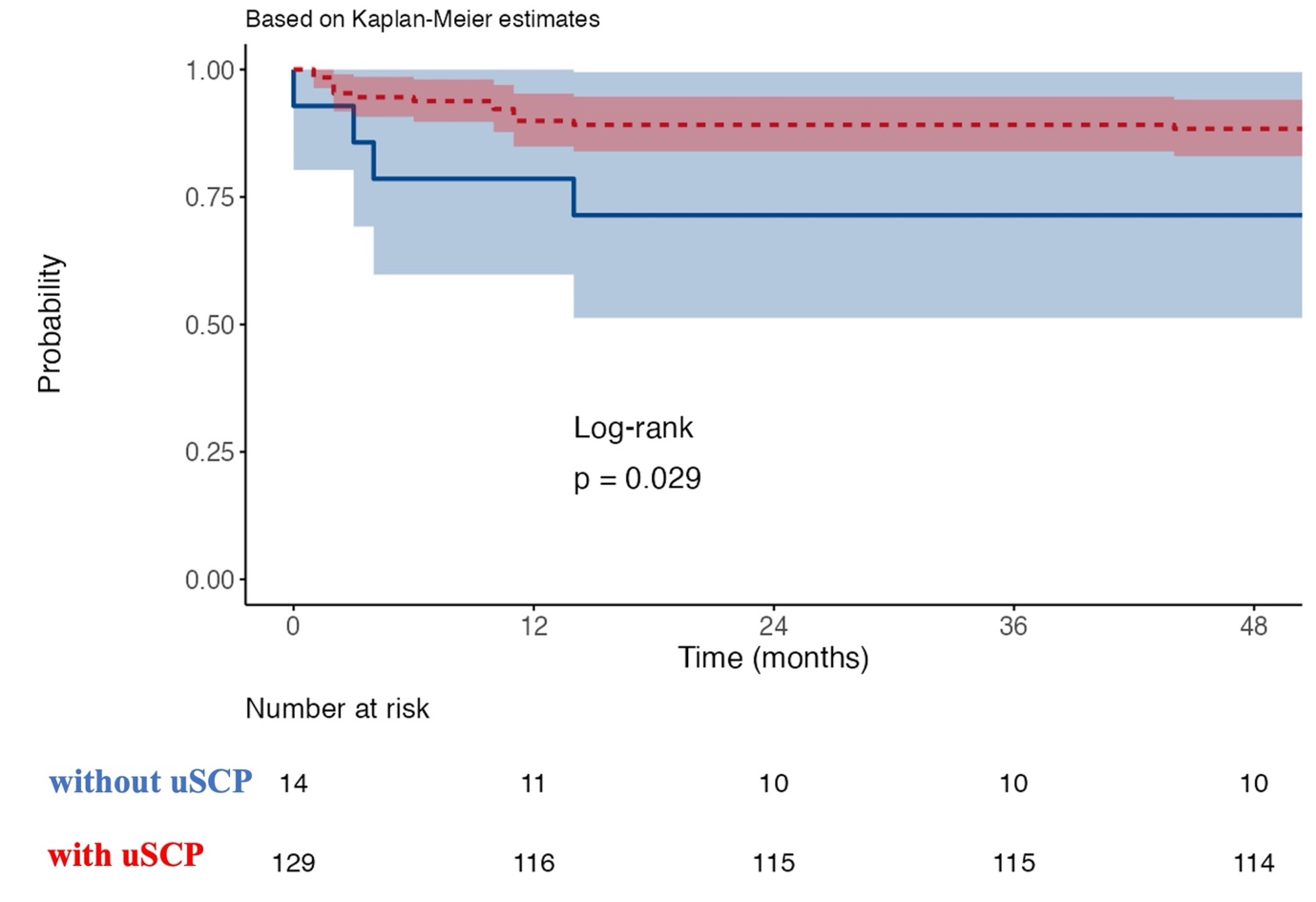
**
